# Optimal parameter identification of synthetic gene networks using harmony search algorithm

**DOI:** 10.1371/journal.pone.0213977

**Published:** 2019-03-29

**Authors:** Wei Zhang, Wenchao Li, Jianming Zhang, Ning Wang

**Affiliations:** Institute of Cyber-Systems and Control, Department of Control and Engineering, Zhejiang University, Hangzhou, China; Maharshi Dayanand University, INDIA

## Abstract

Computational modeling of engineered gene circuits is an important while challenged task in systems biology. In order to describe and predict the response behaviors of genetic circuits using reliable model parameters, this paper applies an optimal experimental design(OED) method to obtain input signals. In order to obtain informative observations, this study focuses on maximizing Fisher information matrix(FIM)-based optimal criteria and to provide optimal inputs. Furthermore, this paper designs a two-stage optimization with the modified E-optimal criteria and applies harmony search(HS)-based OED algorithm to minimize estimation errors. The proposed optimal identification methodology involves estimation errors and the sample size to pursue a trade-off between estimation accuracy and measurement cost in modeling gene networks. The designed cost function takes two major factors into account, in which experimental costs are proportional to the number of time points. Experiments select two types of synthetic genetic networks to validate the effectiveness of the proposed HS-OED approach. Identification outcomes and analysis indicate the proposed HS-OED method outperforms two candidate OED approaches, with reduced computational effort.

## Introduction

In synthetic biology, synthetic gene circuits and networks offer the opportunity to modify behaviors of cellular systems in a controllable and stable way. With well-designed modules or so-called biobricks, it has become feasible to design and build complex DNA circuits that can detect and trigger activities in cells [1]. One purpose of synthetic biology is to design and construct artificial biological systems with certain cellular functions [2, 3]. For instance, a DNA-based chemical oscillator has been constructed to accomplish molecular computing in the future [4]. This prototype DNA oscillator was programmed to generate repeating patterns. Synthetic gene circuits have also been applied for programming cellular functionality [5, 6]. Constructing gene networks involves optimization of biochemical parameters and network topologies. The SynNet method applied a two-step optimization strategy to find the global biochemical parameters [7]. Accurate predictive models are important in aiding the process of designing synthetic biological systems. In order to construct mathematical models, computational approaches are used to infer unknown model parameters using expression data. Emerging genomic data provide necessary information in modeling. The topics discussed in model identification include network structures, functional form of nonlinear dynamics and coefficients that represent reaction rates.

Computational modeling of genetic circuits is not only useful in modular construction of synthetic biological systems, but also is beneficial for exploring the gene regulations mechanisms underlying expression data. Gene circuit modeling has became a powerful tool for synthetic biologists. Multiple types of computational methods have been developed for modeling gene regulatory circuits [8, 9]. Mathematical models of genetic oscillators have been derived to investigate the dynamics of gene regulation [10]. Prior biological knowledge such as sparse interactions and network motifs also play crucial roles in identification [11]. Furthermore, sparse regression approaches have been applied to infer biological networks of nonlinear dynamics [12, 13]. Parameter estimation using single cell expression data will be another promising option. In this study, the fluorescence levels of the reporter protein are used to reflect the expression levels, leading to limited sample size [14]. Compared with experiment-based trail and error approaches, model-based computer aided techniques are able to find the best solution within a defined search space using validated models. However, both solutions are considered to lack efficient predictive capabilities.

Deterministic modeling has the advantage of relatively clear biophysical meanings and computational efficiency. However, current computational approaches and mathematical models are still lacking efficient ability to accurately capture and predict the expression dynamics [15]. In general, such computational modeling tasks are time-consuming, even for medium-size gene circuits [16]. Before identification, the model sets, computational complexity and experimental conditions should be taken into consideration. There are some practical problems, such as the number of experiments and measured time points to guarantee the plausibility and reliability of predictive models. The model complexity, partly reflected by the number of unknown kinetic parameters, directly influence the computational complexity. Some research works have noticed the cost in measuring the expression levels of mRNA and expressed proteins [17]. This analysis is qualitative to some extent. This study tries to analyze the measurement cost quantitatively and introduce a penalty in the cost function. Furthermore, a heuristic optimization is applied to solve the optimization problem.

In order to obtain predictive models of genetic circuits, the principle of maximum entropy was employed to build a minimal model with three constraints [18]. As protein synthesis, degradation and positive feedback have been taken into consideration in the modeling, feedback parameters can be yielded for circuit design. The difficulties in modeling of synthetic biological systems partly lie in structural and parametric uncertainty as well as the lack of efficient information. In order to obtain accurate kinetic parameters, increasing the number of experiments and measured data will be a candidate choice. Meanwhile, the quality of data should not be ignored since it directly influences the accuracy of modeling. Only with high-quality measurements, parameter estimation approaches are possible to obtain reliable parameters of biological networks. Global optimization and nonlinear least square methods are commonly used to infer possible model parameters. In most cases, established parameter estimation methods still have high level of uncertainty in dealing with biochemical networks. Under this circumstance, optimal identification through well designed perturbation experiments become a feasible and promising solution.

As researches always explore approaches to accomplish the model identification of synthetic gene networks in an ACAP(as accurate as possible) style, increasing the amount of measurement is a direct and feasible solution. This expensive and time-consuming solution has limited ability to increase information content of experiments. Accurate models need high-quality measurements, which depend on appropriately designed experiments. Relying only on prior experience is not enough to design input signals in modeling of synthetic gene circuits. In this way, optimal experimental design(OED) provides a feasible way to design input signals, including selection of sampling periods and the number of measured points. The basic principle of OED is to yield informative measurements, thus increasing accuracy of identification. As computational modeling is an importance part in computational systems biology, the topic of OED have been discussed to improve the accuracy of identified models [19]. Measurement noise as well as robust architecture of biological circuits are factors that influence the accuracy and reliability of predictive models. Thus, optimal experiment design is regarded as a powerful tool to minimize the number of experiments needed to infer biological parameters, thus reducing experimental cost. Dynamic models of regulatory networks can be denoted in the form of differential equations [20]. In modeling of biochemical network models, pros and cons of three optimal design approaches have been compared [21]. For computational modeling of biochemical networks, lack of efficient experimental data make it difficult to obtain reliable mathematical models. Under the framework of Bayes estimation, the OED is used to predict which experiments can infer accuracy model parameter distribution [22]. This method applied the k-Nearest Neighbor method to estimate the Jensen Shannon divergence between predictive densities of competing models. For gene network, experimental design is beneficial to reduce the uncertainty in network inference. To collect enough observations for network inference, the resource spent on perturbation experiments is considerable [23]. Well-designed perturbation experiments are necessary to obtain high-quality observations.

The problem of OED is usually converted to an optimization problem that involves the judgment of model quality, which is related the scalar functions of FIM [24]. Several factors such as measured time points, sampling time and efficient information contained in measurements have influence on the modeling quality of synthetic gene circuits. Given a cost function, screening search strategies can be applied to find the suitable input level for a given system. However, this brute search strategy may become ineffective due to huge space of viable points, especially for complex gene networks. Considering this limitation, the optimal input level can be determined in another search strategy. The core problem in optimal identification is to keep balance between estimation accuracy and measurement cost using a well designed cost function. This study applied a modified harmony search algorithm to solve OED problem in modeling gene circuits.

Under the framework of deterministic modeling, the paper proposes an two-stage identification method to obtain model parameters at a low experimental cost. In this method, the model quality is judged by the modified E-optimal criteria and the measurement cost is reflected the number of collected data points. Regarding two factors in computational modeling of gene networks, a cost function with two terms is constructed and minimized in the cost function. The first penalty term represents the derivation between predicted and measured output, while the second penalty term denotes the normalized experimental cost related with the number of collected time points. It is noted that selection of measured time points should consider the complexity of the given system, i.e. the number of unknown parameters. Traditional gradient-based methods hardly accomplish computation of the scalar function of information matrix. This paper applies a optimal identification based on harmony search algorithm to compute FIM-based modified E-optimal criteria as well as the parameter vector of ODE systems. Different with existing parameter estimation methods, the proposed method searches an optimal input level to get most informative observations in the outer loop. Experimental outcomes that involve two kinds of synthetic gene networks illustrate that the proposed optimal identification is able to achieve a tradeoff between estimation accuracy and measurement cost.

## 1 Model identification of gene networks

Under the framework of deterministic modeling, the modeling of gene regulatory networks can be converted to parameter estimation of ordinary differential equation(ODE)-based systems. The task of parameter estimation is to compute parameters of predictive models that predict the system behaviors by extracting information from measurements. There are inherent limitations when applying common estimation approaches in in biological networks. Firstly, empirical parameterizations of the functional form is usually difficult for complex biological networks. Secondly, estimating system parameters typically requires high-quality measured data that is supported by well-designed experiments. Correlation between model parameters is another negative factor that influence estimation accuracy [25]. Other factors such as sampling frequencies and regularization terms in regression also have impact on the performance of deterministic modeling [26].

### 1.1 Parameter estimation of gene networks

System identification approaches need the model set such as ordinary differential equations(ODE) or stochastic differential differential equations(SDE) to describe the expression behaviors for a given system. In this case, low-order approximation of objective function become a feasible solution. In order to describe the expression behaviors of gene networks, the ODE models are defined by Eq (1).

$$\begin{array}{c} \frac{dy}{dt}=f\left(x\left(t\right),u\left(t\right),p\right) \end{array}$$ (1)

where *x*(*t*) denote the observed expression levels of genes, *u*(*t*) is the experimental input signal that can be artificially modified. Parameter vector p* can be estimated by minimizing the cost function that is related with prediction error. As a typical kind of biological networks, gene regulatory networks(GRN) consist of a series of biochemical reactions. Coding regions of DNA will be firstly transcribed as mRNA molecules, then translated to proteins. Expression levels of genes or transcription factors (TFs) are used to estimate unknown kinetic parameters. In deterministic modeling of gene networks, prediction errors between measurements and model parameters are defined by Eq (2).

$$\begin{array}{c} e_{i}=y_{i}-{\hat{y}}_{i} \end{array}$$ (2)

where *y*_*i*_ and ${\hat{y}}_{i}$ denote the measured and predicted output at the measured time *t*_*i*_. In order to estimate the parameters in ODE models for gene networks, gene expression levels often play the role of information source. The operating principle of parameter estimation is mainly based on the minimization of deviation between model prediction and experimental measurements. This study selects the root-mean-square error(RMSE) index to reflect the degree of deviation, which is defined in Eq (3).

$$\begin{array}{c} RMSE=\sqrt{\frac{1}{n}\sum_{i=1}^{n}{\left(y_{i}-{\hat{y}}_{i}\right)}^{2}} \end{array}$$ (3)

where *n* is the number of time points collected. In addition, the residual sum of squares(RSS) is also a measure of the discrepancy between the measured data and the model. As the quality of measurements influence the accuracy of estimation, effects of parameter variations are taken into consideration in the weighted residuals sum square error(wRSSE) index, that is defined in Eq (4).

$$\begin{array}{c} \phi \left(p\right)=\sum_{k=1}^{n}{\left(\frac{y_{k}-{\hat{y}}_{k}}{\sigma_{k}}\right)}^{2} \end{array}$$ (4)

This wRSSE index reflects the degree of uncertainty in measured data to some degree. Gradient-based methods are used to search local minimums of the cost function [27]. According to gradient-based methods, the objective function is approximated by gradient vector, defined by Eq (5).

$$\begin{array}{c} \nabla \phi \left(p\right)={\left(\frac{\partial \phi (p)}{\partial p_{1}},\ldots ,\frac{\partial \phi (p)}{\partial p_{n_{p}}}\right)}^{T} \end{array}$$ (5)

where *p*_*i*_(*i* = 1, …, *n*_*p*_) are inferred components of the parameter vector. Unknown parameter vectors represented as individuals are operated by genetic operations including mutation and selection. Such individuals in the population of heuristic algorithms are regarded as candidate solutions. For instance, solutions are represented as strings of binary numbers in genetic algorithms. Evolutional strategies are relatively efficient in dealing with nonlinear ODEs. With the assumption of positive definite Hessian matrix, unique minimizer for the model can be found by solving the linear system. Thus the parameter vector for next iteration can be defined as Eq (6).

$$\begin{array}{c} p_{k+1}=p_{k}+s_{k} \end{array}$$ (6)

Global optimization methods that minimize *ϕ*(*p*) over all possible values of *p* are hard to find, but local optimization approaches are feasible. To search the optimal parameter vector p*, evolutionary algorithms have been widely applied in the optimization problem.

### 1.2 Sensitivity analysis of gene networks

The initial purpose of sensitivity analysis is to find those parameters that influence system dynamics significantly. Sensitivity analysis also plays a special role in analyzing practical identifiability and uncertainty assessment. For the given system *y* = *f*(*x*, *p*, *t*), changes of systems states *x*(*t*) depend on changes of model parameters *p* around a reference point *s*(0), described by Eq (7).

$$\begin{array}{c} s\left(t\right)=\frac{\partial x(t)}{\partial p} \end{array}$$ (7)

Regarding the parameter space, time-dependent linear approximation of parameter sensitivity behaviors can be captured by finite difference approximation. Using augmented dynamic ODE system, implicit computation of the sensitivity matrices for gene network are calculated as Eq (8).

$$\begin{array}{c} \frac{ds(t)}{dt}=J_{x}\left(t,p_{0}\right)s\left(t\right)+J_{p}\left(t,p_{0}\right) \end{array}$$ (8)

Where *J*_*x*_(*t*, *p*_0_) = ∂*f*(*x*, *p*, *t*)/∂*x* and *J*_*p*_(*t*, *p*_0_) = ∂*f*(*x*, *p*, *t*)/∂*p* are Jacobian matrices evaluated at the parameter values *p*_0_. With the assumption of Gaussian approximation and *Q* = *I*, the Hessian matrix $H_{ij}\left(p\right)=\nabla_{ij}^{2}\phi \left(p\right)$ of the objective function *ϕ*(*p*) can be rewritten in terms of parameter sensitivities, denoted by Eq (9).

$$\begin{array}{c} H_{ij}\left(p\right)={\left(S\left(p\right)S\left(p\right)\right)}_{ij}^{T} \end{array}$$ (9)

where *S*(*p*) denotes a matrix of time-dependent blocks *s*_*ij*_(*t*_*k*_) = ∂*x*_*i*_(*t*_*k*_)/∂*p*_*j*_. Existence of linearly dependent columns of the sensitivity matrix *S*(*p*) will lead to non-unique solutions of *p*. That indicate part of model parameters are unidentifiable at a specific reference point *p*_0_ in parameter space. Correlations between two column vectors *S*_.,*i*_, *S*_.,*j*_ of the sensitivity matrix *S* are described by Eq (10).

$$\begin{array}{c} corr\left(S_{.,i},S_{.,j}\right)=\frac{cov(S_{.,i},S_{.,j})}{\sigma \left(S_{.,i}\right)\sigma \left(S_{.,j}\right)} \end{array}$$ (10)

where *σ*(*S*_.,*i*_), *σ*(*S*_.,*j*_) represent the covariance between the *i*-th and *j*-th columns of *S*. For two linearly dependent columns, the correlation index is |*corr*(*S*_.,*i*_, *S*_.,*j*_)| = 1. Threshold for correlation between two model parameters is related with pairwise correlations.

### 1.3 FIM-based estimation accuracy analysis

After obtaining the optimal parameter vector $\hat{p}$, goodness-of-fit(GOF) and estimation accuracy will be analyzed in subsequent evaluation. As true kinetic parameters p* are unknown, it is crucial to judge the accuracy of estimated parameters and the degree of deviation. Assume fixed model structure, the observed data become mixed with the measurement noise, that is described by Eq (11).

$$\begin{array}{c} x^{M}\left(t_{i}\right)=x\left(p,t_{i}\right)+\epsilon \left(t_{i}\right) \end{array}$$ (11)

With the assumption of a normally distributed random variable on measurement error, the resulting residuals for each measurement are given as Eq (12).

$$\begin{array}{c} e\left(x,p,t_{i}\right)∼N\left(0,\sigma_{i}^{2}\right) \end{array}$$ (12)

With the objective function $\phi \left(p\right)=\sum_{i=1}^{N}e_{i}^{T}Q_{i}e_{i}$, the measurement variance can be applied to construct weight matrix *Q*. Generally, there are two options in values of weight matrix *Q*. The choice of *Q* = *I* corresponds to equal weight for errors, regardless of measurement accuracies. The situation *Q* = *C*^−1^ indicates weighting based on the inverse of the measurement covariance matrix *C*. In the second situation, standard deviations of measurements appear on the diagonal positions. According to the principle of goodness-of-fit(GOF), statistics for *ϕ*(*p*) should follow *χ*^2^ distribution with *r* degrees of freedom, where *r* equals to the number of data points minus that of estimated parameters. With an assumption of norm distribution, i.e. *X*_*i*_ ∼ *N*(*μ*, *σ*^2^), the average value is denoted as $\sum_{i=1}^{N}\frac{X_{i}}{N}$. Additionally, the sampling distribution is defined as Eq (13).

$$\begin{array}{c} S^{2}=\sum \frac{{\left(X-X_{i}\right)}^{2}}{N-1} \end{array}$$ (13)

This sampling distribution associated with the sample variance follows a *χ*^2^ distribution with *N* − 1 degrees of freedom, i.e. $S^{2}\left(N-1\right)/\sigma^{2}∼\chi_{n-1}^{2}$. If a new statistic *Z* is constructed as $Z=\left(X-\mu_{X}\right)/\sigma_{X}^{2}$, then this statistic index implies the chi-squared distribution *χ*^2^(*k*), denoted by Eq (14).

$$\begin{array}{c} \sum_{i=1}^{k}Z^{2}∼\chi^{2}\left(k\right) \end{array}$$ (14)

In computational modeling of gene networks, large-scale models and scarce experimental data will inevitably lead to limited observability of states as well as uncertainty in parameter estimation. Under the Gaussian-Markov assumption, the least squares estimator can be regarded as un-biased estimators, in which the variance is minimized. With the optimal parameter vector p*, the inverse matrix of variance for p* is called the information matrix.

After solving the time-dependent ODE equations, the sensitivity matrices are computed as $S_{t_{i}}=\partial c/\partial p$ at time points *t*_*i*_(*i* = 1, …, *N*). Under the Gaussian assumption, the Fisher information matrix can be calculated as Eq (15).

$$\begin{array}{c} F\left(p^{*}\right)=\sum_{i=1}^{N}{\left(\frac{\partial c}{\partial p}\right)}^{T}C^{-1}\left(t_{i}\right)\left(\frac{\partial c}{\partial p}\right) \end{array}$$ (15)

Given input-output data, the FIM represents a measures of the information content with regard to the parameters *θ* in the model. If the measurements for *c*_*p*_(*t*_*i*_) and *c*_*s*_(*t*_*i*_) are independent, then the covariance matrix of measurements consist of diagonal elements, described by Eq (16).

$$\begin{array}{c} c_{m,n}\left(t_{i}\right)=\sigma_{m}^{2}\left(t_{i}\right) \end{array}$$ (16)

In Eq (16), *cr*_*jj*_ denotes the diagonal elements of the inverted FIM and $\sigma_{m}^{2}\left(t_{i}\right)$ represent the variance of measured state variables *m* at time point *t*_*i*_. Assume that Gaussian noise in the measured data, the FIM is given by Eq (17).

$$\begin{array}{c} F\left(p^{*}\right)=\sum_{i=1}^{N}S{\left(t_{i};p^{*}\right)}^{T}diag\left(\frac{1}{\sigma_{i}^{2}}\right)S\left(t_{i};p^{*}\right) \end{array}$$ (17)

Where *S*(*t*_*i*_; p*) denote sensitivity matrix for p*, and $diag(1/\sigma_{k}^{2})$ represents the measurement covariance. The diagonal elements of covariance matrix *C*(*t*_*i*_) should be positive. Parameter uncertainty region can be further determined by eigenvectors and inverse eigenvalues of the FIM. In order to analysis estimation accuracy in deterministic modeling, the parameter uncertainty region is investigated using the information matrix *F*(p*). In general, small eigenvalues of *F*(p*) indicate large uncertainty. If the calculated *F*(p*) is non-singular, then the Crámer-Rao lower bound for the variances of estimated parameters can be determined according to Eq (18).

$$\begin{array}{c} \sigma_{j}\geq \sqrt{F{\left(p^{*}\right)}_{i,i}^{-1}} \end{array}$$ (18)

where $F{\left(p^{*}\right)}_{i,i}^{-1}$ are diagonal elements of information matrix. The framework of estimation accuracy analysis using FIM-based optimality is designed considering both model accuracy and measurement cost. Given measured expression levels of expressed proteins, the locally optimal kinetic parameters are inferred through curve fitting. Sensitivity matrices for model parameters are calculated to construct FIM.

## 2 Optimal identification of gene networks

Considering complex regulations between network components, it is beneficial to perform optimal experimental design to obtain measurements with maximized information content. Informative measurements are useful in enhancing the accuracy of parameter identification. Generally, experimental design can be performed based on statistic criterions that are closely related with covariance matrices of the selected models. The OED problem for gene network inference have been investigated in order to obtain mathematical models with high credibility [28–30]. For simulation-based OED, the basic purpose is to minimize the uncertainty bounds on the estimated parameters [31, 32]. Computational approaches play a crucial role in reducing the uncertainty of structure identification and parameter estimation. This section will propose a novel framework that pursue a balance between estimation accuracy and measurement costs in modeling synthetic gene networks.

### 2.1 Basic framework of optimal experiment design(OED)

For deterministic modeling of synthetic gene circuits, major factors that influence the accuracy of estimation include but are not limited to information content of measurement, the number of time points, the sampling periods of output. There are several optimal principles that guide the process of optimal design. The D-optimality principle tries to minimize the covariance matrix of parameter estimate, which correspond to maximizing the determination of information matrix. The A-optimality seeks to minimize the trace of the inverse of the FIM. This A-optimality criterion results in minimizing the average variance of estimated regression coefficients. As for the commonly used D-optimality, this principle maximize the determinant of the FIM and results in maximizing the differential Shannon information content of the parameter estimation [33, 34]. The OED problem can be solved by maximizing measures of FIM, i.e. ζ* = *argmax*Φ(*FIM*(*θ*, *ζ*)), where *ζ* is a function of input level and sampling periods etc. The measurement set selection design can be converted to an optimization problem $min\sigma^{2}{\left(\Sigma_{i=1}^{n}\lambda_{i}S_{i}^{T}S_{i}\right)}^{-1}$, where λ_*i*_ is an integer weight that is either 0 or 1.

Another design principle is based on E-optimality, where the minimum eigenvalue of FIM is maximized. This study applies the modified E-optimal criteria as the quantitative index to evaluate the constructed mathematical models of gene networks. The modified E-norm of the FIM is defined as the ratio of the maximum eigenvalue of the FIM by its minimum eigenvalue [35, 36]. This modified E-optimal criteria is defined by Eq (19).

$$\begin{array}{c} \|F\left(t_{f},\theta \right)\|=\frac{max\lambda_{F}}{min\lambda_{F}} \end{array}$$ (19)

where λ_*F*_ represent the eigenvalues of information matrix $F(t_{f};\hat{\theta })$, *t*_*f*_ represents the duration time of perturbation experiments. Information matrix are calculated based on best available estimated parameter vector *θ* instead of true value θ*. This modified E-optimal criteria can be further used a useful index to determine the appropriate input level that is likely to provide highly informative measurements.

As the sample sizes of measurements are usually limited for regulatory networks due to experimental cost [37], this paper applies an optimization method that reduce experimental cost without loss of modeling accuracy. Both quality and experimental cost have been taken into consideration in the cost function. The proposed OED method search an appropriate sample size by solving the optimization problem, shown by Eq (20).

$$\begin{array}{c} J\left(n_{m}\right)=argmin_{n_{m}}\left(\sum_{i=1}^{n}{\left(y_{i}-{\hat{y}}_{i}\right)}^{2}+\lambda \frac{n_{m}-n_{p}}{n_{m}}\right) \end{array}$$ (20)

where *n*_*m*_ and *n*_*p*_ represent the number of measured time points and parameters. In the proposed cost function, the first penalty term represents estimation accuracy, with a modified E-optimal criteria. The second penalty term corresponds to the normalized measurement cost, which is related with the number of measured time points. The second penalty term is motivated by the degree of freedom in *χ*^2^ distribution. Since the combination of two monotonic functions lead to a unimodal function, the trajectory of cost function is expected to firstly decline and then increase after reaching the minimal point.

### 2.2 Harmony search-based OED

As a kind of nature-inspired optimization, harmony search(HS) algorithm shares certain characters with other evolution strategies algorithms such as genetic algorithms. The basic idea of harmony search algorithm is to find a vector *x* that minimizes a given objective function [38]. Standard HS algorithm begins with generating *HMS* random vectors and stores these vectors in harmony memory denoted by *HM*. New candidate solution vectors *x*_*new*_ are generated to replace the worse vector in *HM*. As initial algorithm parameters, global variables including the number of variables *v*, the maximum of iterations *uIter*, *pIter*, harmony memory size *HMS* and harmony consideration rate *HMCR* are defined. The pseudo code of HS-based OED is described by Algorithm 1.

There are two iteration loops in the diagram, where the outer loop is responsible to find the best input level u* and the inner loop update the best model parameter estimation p*. In Algorithm 1, *lb*, *ub* represent the lower and upper bound for the values of *HMu*, *HMp*. The *cfun*1, *cfun*2 represent the cost function of inner and outer iteration loop respectively.

**Algorithm 1**: Pseudo code of HS-based optimal experimental design algorithm.

**Input**: Choose input *u*0 based on prior knowledge.;

1 Initialization of HS-OED algorithm: *HMu*,*HMp*,*PAR*,*HMCRu*,*HMCRp*,*NV AR*;

2 Estimate parameter vector *p*0 using *u*0;

3 **for** *i = 1 to uIter* **do**

4  initialize *HMu*;

5  *HMu*(1,:) = $u_{best}^{*}$;

6  *HMu*(2:end,:) = *lb*+(*ub*-*lb*)*rand();

7  Compute *cfun*1(*u*, *p*) of every *u* in *HMu* and load them into fitness array;

8  Compare fitness array to find best input;

9  Update *HMu* and fitness array;

10  Update $u_{best}^{*}$ as best input;

11  **for** *j = 1 to pIter* **do**

12   initialize *HMu*;

13   *HMp*(1,:) = $p_{best}^{*}$;

14   *HMp*(2:end,:) = *lb*+(*ub*-*lb*)*rand();

15   Compute *cfun*2(*u*, *p*) for every parameter in *HMp* and load to *fit*2;

16   Compare *fit*2 array to determine the best parameter vector p*;

17   Update *HMp* and *fit*2 array;

18   Update $p_{best}^{*}$ as the current best parameter vector;

19  **end**

20 **end**

**Fig 1:**
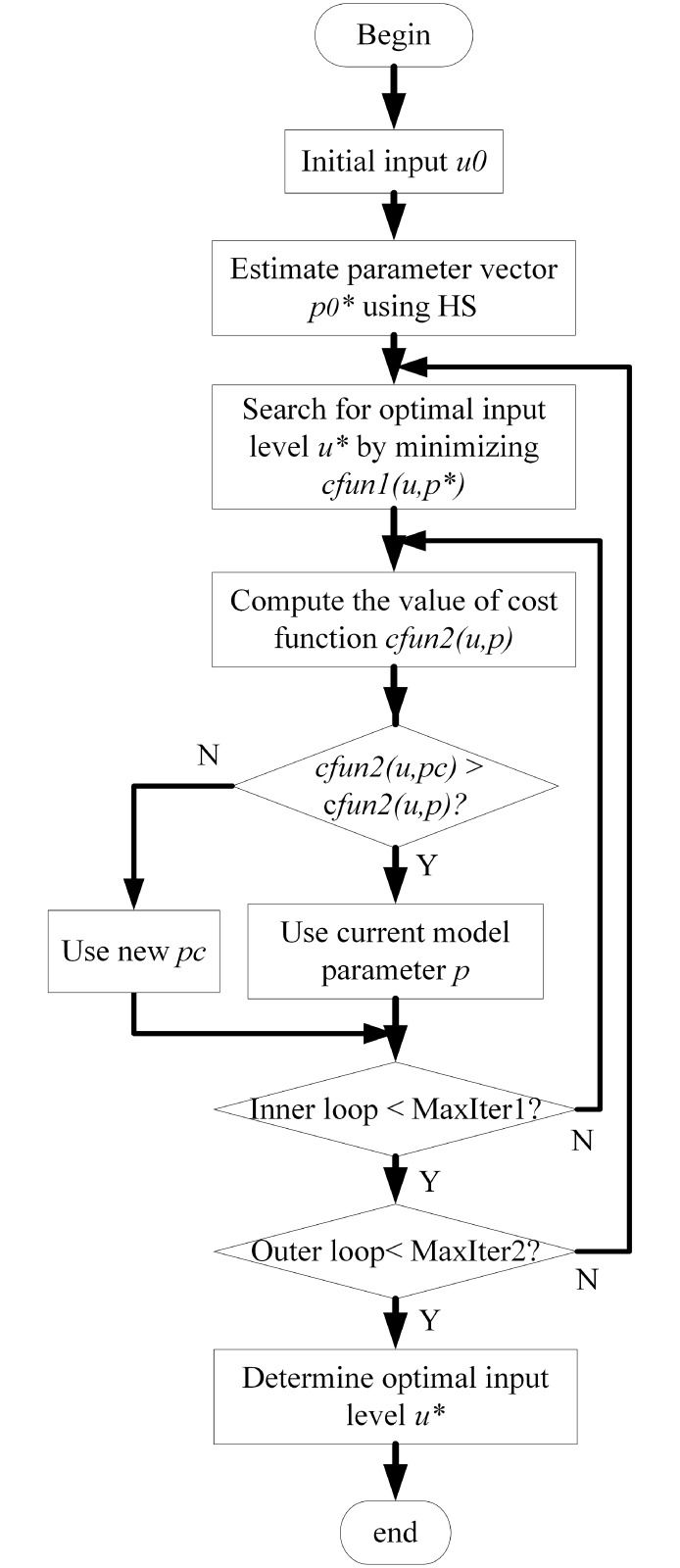
The flow chart of harmony search-based OED using a double nested loops. The inner loop updates the parameter vector based on an initial model parameter vector and the outer loop aims to determine the optimal input level u* by minimizing the cost function.

**Output**: Optimal input level u*, parameter vector p*;

In the HS-based OED framework, *u*_*best*_ and $p_{best}^{*}$ are used to find better parameter vector *p*_*best*_. The proposed HS-based OED determines the optimal input level u* in the outer loop and computes the model parameter p* in the inner loop. When the inner iteration obtains a candidate parameter vector *p*_*c*_, it is necessary to compare the value of *cfun*1(*u*, *p*_*c*_) with that of current best parameter vector p*. When the condition *cfun*1(*u*, *p*_*c*_) < *cfun*1(*u*, p*) is satisfied, the model parameter vector will be updated as p* = *p*_*c*_. In this way, the second stage of iteration optimization begins searching the optimal parameter vector u* with optimal input level p*. This two stage optimization framework is described by Fig 1.

When brute screening strategy is applied to find a suitable input level, the screening sizes and steps should be carefully selected. Such screening strategy is relatively inefficient and restricted to a fixed combination of possible input levels. Compared with the screening search strategy, this study applies random search strategy which is able to find an flexible input level rather than selecting one fixed input value. Another advantage of this random search strategy is that the optimal parameter vector of a given system is estimated at the same time.

## 3 Experimental outcomes and analysis

The main purpose of deterministic modeling based OED is to offer an suitable input signal u* for two types of genetic circuits. Kinetic model parameters are estimated and evaluated from the view of error indexes. Two types of engineered gene circuits with mathematical models are selected as benchmarks [39]. Each system is described by a set of ODEs that describe biochemical reactions governing protein species. Parameter estimation for synthetic gene networks are performed in two stages: initial estimation and parameter refinement. Initial parameter vectors are computed based on prior information and biological experience such as the response time of genetic circuits. The refinement pursues parameters with higher accurateness with the initial parameter vector.

### 3.1 Unbuffered synthetic gene networks

In the unbuffered system, the chemical species *SKN*7*m* activates expression of green fluorescence protein(GFP) from the synthetic promoter *P*_*TR*−*SSRE*_. With the small molecule doxorubicin *DOX* as input, the reactions considered in the unbuffered system include the *DOX* activated production of *SKN*7*m*, the binding and unbinding of *SKN*7*m*, the activated production of reporter protein(GFP) and degradation of all species. Define *C*_*m*_ as the complex formed between *SKN*7*m*, the simplified reactions are described as followings:

$$\begin{array}{c} \begin{array}{c} SKN7m+p\overset{k_{on}}{\underset{k_{off}}{⇌}}C_{m}\\ \;C_{m}\overset{\delta_{c}}{\to }SKN7m+p \end{array} \end{array}$$ (21)

where *δ*_*c*_ denotes the degradation rate of *C*_*m*_. Established modeling approaches choose periodic inputs to maximize the steady state peak-peak amplitude percent error between the loaded and unloaded trajectory of output protein *GFP*. Furthermore, multiple types of inputs including two step and three square inputs are used to induce the response of unbuffered gene networks and to provide input-output data for parameter identification. Error indexes of wRSSE and RMSE under different types of input signals are computed in Table 1.

**Table 1:** Error indexes of *k*_*on*_, *k*_*off*_ in modeling unbuffered gene network. The wRSSE and RMSE indexes are selected to judge the performance of parameter estimation. The title Step *i*(*i* = 1, 2) represent the single and double inputs with the same concentration 20 *μM*. The columns *T*_100_, *T*_200_, *T*_400_ denote the periodic inputs with periods *T*_1_ = 100min,*T*_2_ = 200min and *T*_4_ = 400min.

Errors	Step 1	Step 2	*T*_100_	*T*_200_	*T*_400_
wRSSE	3.914×10^−9^	2.403×10^−7^	4.684×10^−6^	5.332×10^−6^	8.837×10^−6^
RMSE	6.995×10^−6^	5.481×10^−5^	2.419×10^−4^	2.581×10^−4^	3.323×10^−4^

**Fig 2:**
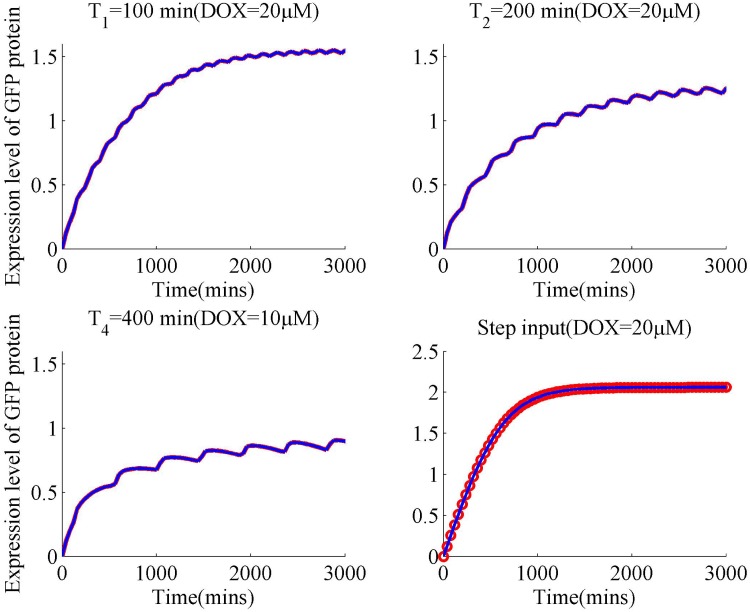
Curve fitting of unbuffered gene circuit using four types of input signals. The periods of first three square inputs are 100,200 and 400 mins respectively. In order to get stable response curve, the simulation time is set as 3000 mins.

In the parameter setting part of harmony search algorithm, the number of variables is settled as *n*_*p*_ = 3, the harmony memory size *HMS* = 15, the harmony consideration rate *HMCR* = 0.2. Matrices including *HMu*, *fitu* are initialized to store relevant indexes, where *fitu* represents a fitness vector related with optimal input. The first step of optimal identification is to compute the parameter vector using measured input-output data. With the computed vector *p*, response curves of unbuffered gene networks using four types of inputs including single step up and three periodic inputs are fitted, shown in Fig 2.

In Fig 2, the oscillatory periods for three square inputs are 100, 200 and 400 mins. Fitted responses of the unbuffered system are basically consistent with the measured output trajectories. This indicates that estimated parameters *k*_*on*_ and *k*_*off*_ are able to capture part of response dynamics under these circumstance.

Further estimation take all 9 kinetic parameters into consideration. Since incoherent noise may have negative influence on parameter estimation, parameter estimation is performed in two groups: simulation(S) and experimental(E) group. Deterministic modeling of unbuffered systems with 9 kinetic parameters are performed using a step input and four periodic inputs. Similarly, the weighted RSSE(wRSSE) and RMSE indexes under different types of input signals are computed and compared in Table 2.

**Table 2:** Error indexes of 9 kinetic parameters in modeling unbuffered gene network. The wRSSE and RMSE indexes are selected to evaluate the performance of parameter estimation. The column denoted Step 1 represents the single and double inputs with the same concentration 20 *μM*. The columns *T*_150_, *T*_200_, *T*_250_ and *T*_500_ denote the periods of square inputs are 150, 200, 250 and 500mins. The symbol # indicates the shortage of relevant tests.

Errors	Step 1	*T*_150_	*T*_200_	*T*_250_	*T*_500_
wRSSE(S)	0.0854	0.0006	0.0015	0.0019	0.0070
RMSE(S)	0.1660	0.0416	0.0679	0.0767	0.1476
wRSSE(R)	#	0.0047	0.0063	0.0122	0.0079
RMSE(R)	#	0.1215	0.1407	0.1951	0.1598

**Fig 3:**
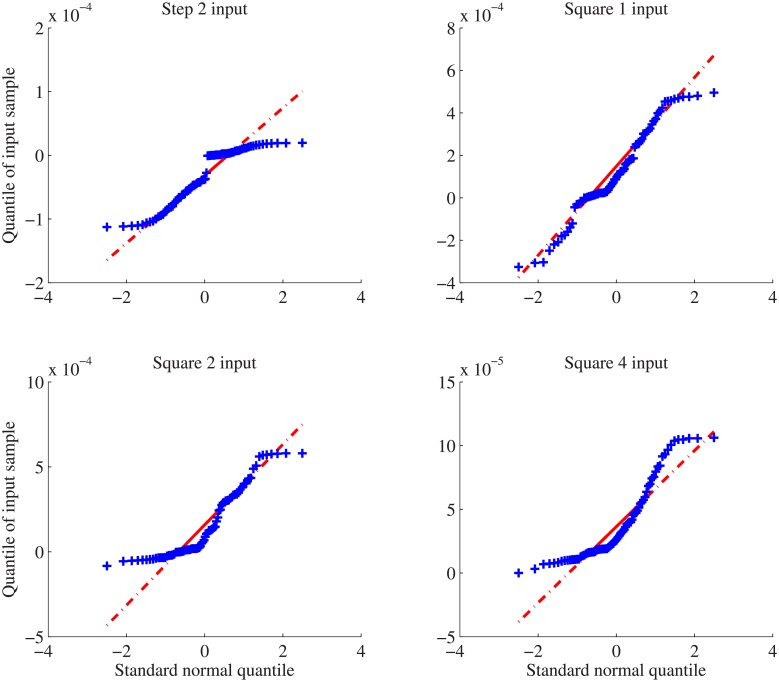
The quantile-quantile(QQ) plots for estimated parameters of unbuffered gene networks using step and periodic inputs. These QQ plots for unbuffered systems show similar patterns observed in error indexes. Squared inputs are likely to reduce the deviation between estimated parameters from the true values.

Since the step test in experimental group provides low-quality measurement, the realistic group only discusses the errors using periodic inputs. Both wRSSE and RMSE indexes fluctuate depending on selection of input signals. This phenomenon indicates that selection of input signals will influence the deviation of estimated parameters from the true values. One of possible reasons is that degrees of perturbation posed to the system are in different levels. Among four square inputs, error indexes obtained by Square 4 that has the least number of measured data points are highest in two groups. During deterministic modeling, the values of wRSSE indexes are generally lower than that of RMSE. To evaluate the effectiveness of estimated parameters, the quantile-quantile(QQ) plots are introduced. When a set of observations are approximately normally distributed, a normal QQ-plot of the observations will lead to an approximately straight line. In this study, QQ-plot is used to compare the estimation error with normal distribution. In numerical simulation, Step 2 represent double step inputs, while square inputs denoted by Square 1,2,4 correspond to the periods of 100, 200 and 400(mins). To visualize the accuracy of estimated model parameters, the quantile-quantile(QQ) plot of Step 2 and Square 1 inputs are compared in Fig 3.

**Fig 4:**
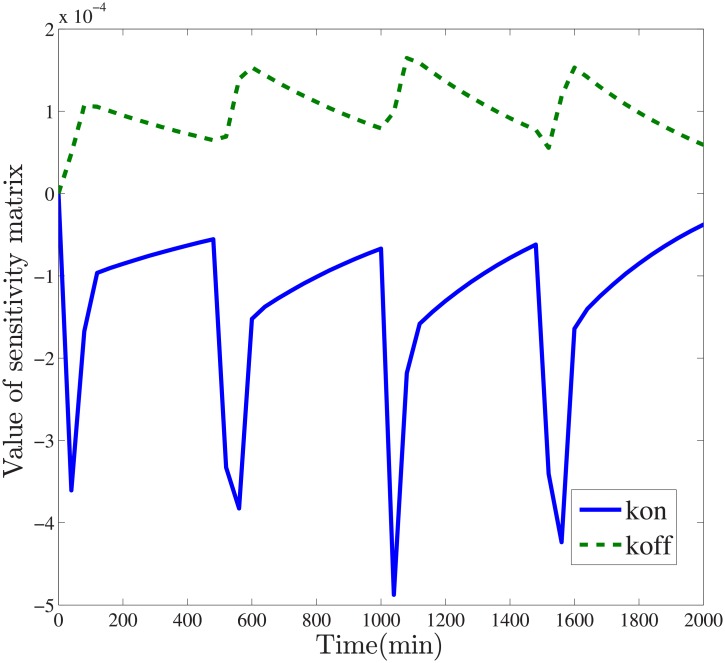
Sensitivity analysis of unbuffered gene circuits. The kinetic parameter *k*_*on*_ has negative sensitivity while the degradation rate *k*_*off*_ has positive sensitivity. The values of sensitivity matrices exhibit periodic changes.

The QQ-plots of estimated parameters for the unbuffered system reflects the deviation of estimated errors against that of standard normal quantile. In Fig 3, the deviation under Square 1 input is smaller than that of other three input signals. Subsequent sensitivity analysis computes the variance for estimated parameters, especially key kinetic parameters. Sensitivity analysis aims to detect those parameters that have significant influence on the response behaviors of a given system, depicted in Fig 4.

In Fig 4, the parameter *k*_*on*_ has negative sensitivity while *k*_*off*_ has positive sensitivity. That means changes in the value of kinetic parameters *k*_*on*_, *k*_*off*_, have opposite impacts on the response of *GFP* protein. The quantitative influence of *k*_*on*_ is slightly higher than that of *k*_*off*_. With the computed Fisher information matrix, the Crámer-Rao lower bound(CRLB) for *k*_*on*_ and *k*_*off*_ are computed as 0.687 and 0.0415 respectively, illustrating that parameter uncertainty for production rate *k*_*on*_ is higher than that of the degradation rate *k*_*off*_.

Another important task of optimal identification is to provide a low-cost solution without much loss of model accurateness. Measurement cost, which is reflected in the number of inferred parameters, is expected to be high for complex systems. When the number of measured points go beyond a specific level, the benefit brought by increasing the sample size become limited while the cost keeps increasing. The optimal identification approach uses a modified harmony search(HS) method to select a suitable sample size. Selection of measured output points *n*_*m*_, should also consider the complexity of a given system, that is related with the number of unknown parameters *n*_*p*_. When the value of *n*_*m*_ exceeds a specific level, the benefit of increasing the amount of measured time points in enhancing the modeling accuracy will be limited.

### 3.2 Buffered synthetic gene networks

**Fig 5:**
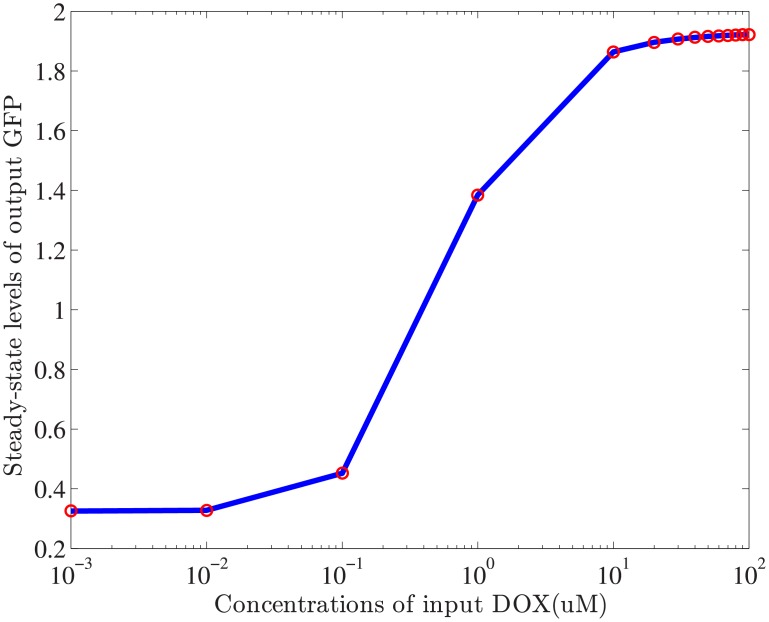
The input-output relations between input *DOX* and output GFP for buffered genetic circuit. Optimal experiment design requires a suitable input level to stimulate the system behaviors. In the numerical simulation, the variation range of input *DOX* concentration is selected as [10^−3^,10^2^]*μ*M. The output-input ratio for buffered gene circuit decreases as the concentrations of input *DOX* increases, and remains stable a level of 0.0195.

In the buffered gene networks, an additional driver module was introduced in the buffered gene networks to eliminate the retroactivity and to improve the response dynamics of regulatory systems. Similar with the unbuffered system, the small molecule *DOX* induces expression of protein *STAT*5 − *HKRR* and activator of transcription 5(STAT5). Buffered genetic circuits accomplish cellular functions based on a series of biochemical reactions, which include DOX activated production of STAT5-HKRR from promoter, SKN7 activated production of reporter protein. Phosphorylation of STAT5-HKRR and YPD1/SKN7 phosphotransfer reactions can be described as the followings:

$$\begin{array}{c} \begin{array}{c} STAT5-HKRR^{*}+YPD\overset{k_{1}}{\underset{k_{2}}{⇌}}STAT5-HKRR+YPD1^{*}\\ SKN7+YPD1^{*}\overset{k_{3}}{\underset{k_{4}}{⇌}}SKN7^{*}+YPD1\\ SKN7^{*}+YPD1^{*}\overset{k_{3}}{\underset{k_{4}}{⇌}}SKN7^{**}+YPD1 \end{array} \end{array}$$ (22)

where *SKN*7** denotes doubly phosphorylated *SKN*7, which activates expression of reporter *GFP* from the synthetic promoter *P*_*TR*−*SSRE*_ and also binds plasmid-encoded load sites. In unbuffered systems, *SKN*7*m* binds promoter directly, while *SKN*7 in this buffered system needs activation by a series of phosphotransfer reactions. Input concentrations of small molecule *DOX* will change the response behaviors of genetic circuits. When the concentration of step signal increases, the output level of reporter protein *GFP* increase as a consequence, shown in Fig 5.

**Fig 6:**
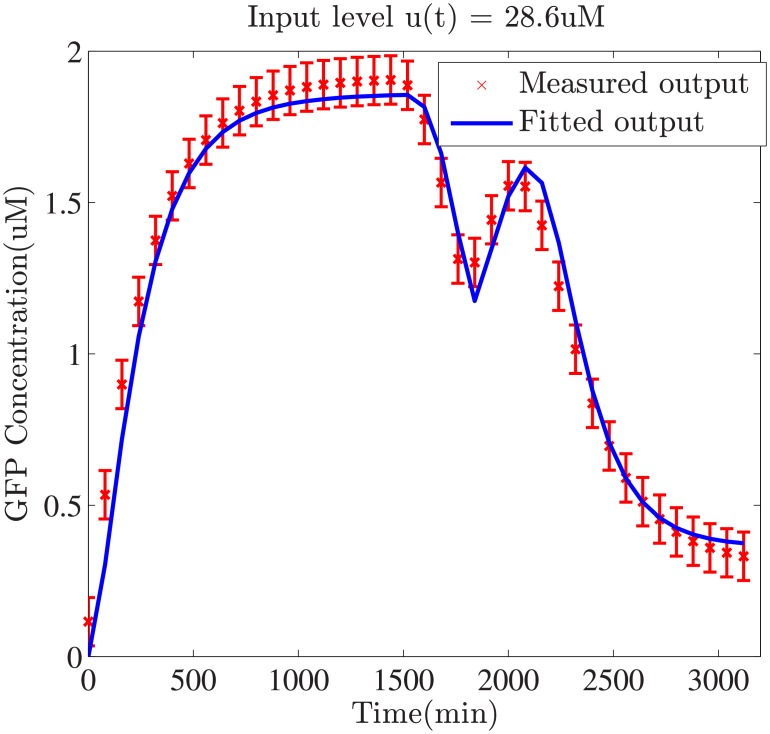
Curve fitting for output of buffered gene networks. In order to validate the effectiveness of estimated parameters, an additional step up signal is introduced to stimulate the response behavior of buffered systems.

In Fig 5, the value of output-input ratio that can be also regarded as the gain of gene circuits, changes in the interval [1.95 × 10^−2^, 350]. Low-level magnitude of input signal contain limited energy to perturb the system dynamics while high level of input push the system to a saturation condition. When the magnitude of input exceeds a threshold, the expression level of reporter protein declines to increase and keeps a stable level. It is important to choose a suitable level of input for subsequent parameter refinement. Instead of grid search, a novel two-step search strategy is applied to determine the optimal level of input. After double nested iterations using harmony search(HS) algorithm, the optimal level of input is selected as 28.6 *μ*M and the model parameter p* is inferred. Using this optimal input level, the comparison of predicted and measured output of buffered gene network using estimated parameter p* are shown in Fig 6.

In the first step, initial model parameter vector *p*_0_ in ODEs of buffered gene networks are computed by minimizing the error index between the predicted and measured output. From Fig 6, the trajectories of predicted output match the measured output, revealing that initial parameter vector capture output dynamics to some extent. Multiple squared inputs are used to validate the effectiveness of parameters under various experimental conditions. The magnitude of testing inputs is set as 20*μM* and the oscillatory periods of square waves are selected as 100,200,300,400,500 (min) respectively.

**Fig 7:**
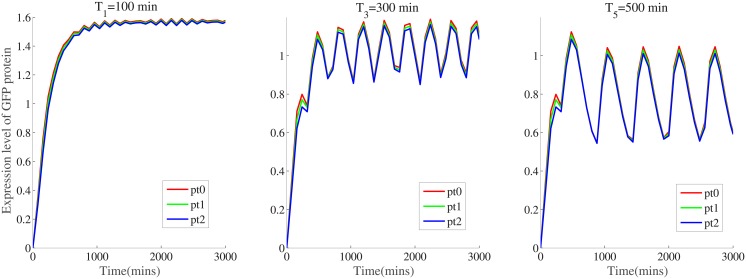
Comparison of predicted output trajectories and measured output GFP concentrations under three square inputs. The periods for three square inputs are 100,300,500 mins. The legends pt0, pt1 and pt2 correspond to the experimental conditions of unloaded(model+0x), single loaded(model+x) and double loaded(model+2x).

In addition, response behaviors of the buffered system are also influenced by adding load plasmids that are regarded as genetic loads. In numerical simulations, three levels of genetic loads are introduced to evaluate the robustness of inferred model parameters. The load variants are encoded on high-copy 2*μ* yeast plasmids, with unloaded(model+0x), single loaded(model+x) and double loaded(model+2x) additional copies of *P*_*TR*−*SSRE*_ [40]. Such genetic loads lead to reversible binding reactions and slow down the increase of free *SKN*7*m* that activates the expression of reporter protein *GFP*. Under these circumstances, the predicted trajectories of expressed *GFP* are compared with that of the measured output, shown in Fig 7.

According to the principle of persistent excitation, square input signals are considered to excite the system dynamics more sufficiently than step signals. It is observed from Fig 7 that response curves under the conditions of model+0x, model+1x and model+2x are approximately the same trajectories. Under three levels of loads, the response curves of buffered system show limited attenuation. In the part of parameter refinement, estimated parameter vector p* reflects the desired behavior of buffered systems. Subsequent analysis will evaluate the accuracy of parameter vector numerically.

Under regulation of multiple periodic inputs, the response curves of buffered gene networks exhibit oscillatory behaviors with different periods. With measured input and output data, unknown parameters of the ODE model can be acquired by estimation algorithms. After obtaining the parameter vector p* using simulated and experimental datasets, two kinds of error indexes including wRSSE and RMSE indexes are calculated. Compared with single step signal, double input signal is introduced in the simulation group. In simulation(S) and experimental(E) group, two step inputs and four square inputs are introduced to perturb system dynamics. Signal periods Square *i*(*i* = 12, 3, 4) in the simulation group are selected as 150,200,250 and 500 minutes, that are consistent with the settings of inputs in realistic experiments. Two kinds of error indexes are computed and depicted in Table 3.

**Table 3:** Performance evaluation indexes of deterministic modeling for the buffered gene network. The columns *T*_150_, *T*_200_, *T*_250_ and *T*_500_ denote the periods of square inputs are 150, 200, 250 and 500 minutes. The symbol # indicates the lack of measured data with efficient quality.

Errors	Step 1	Step 2	*T*_150_	*T*_200_	*T*_250_	*T*_500_
wRSSE	0.0024	0.0276	0.0021	0.0019	0.0022	0.0014
RMSE	0.0271	0.0186	0.0813	0.0775	0.0838	0.0660
wRSSE	#	#	0.0066	0.0091	0.0103	0.0048
RMSE	#	#	0.1436	0.1689	0.1853	0.1223

It is observed from Table 3 that double input signal Step 2 is able to obtain relatively low error indexes, than that obtained by single step input Step 1 in simulation. This phenomenon indicates that double step input can improve the estimation accuracy by increasing the degree of perturbation. Meanwhile, error indexes of the buffered system are slightly higher than that of unbuffered system, reflecting robust of the proposed estimation algorithm. Among four square inputs, the periodic input *T*_2_ = 200*min* obtains the lowest estimation error, showing that oscillatory periods of square inputs is another potential factor that influence the accuracy of deterministic modeling.

**Fig 8:**
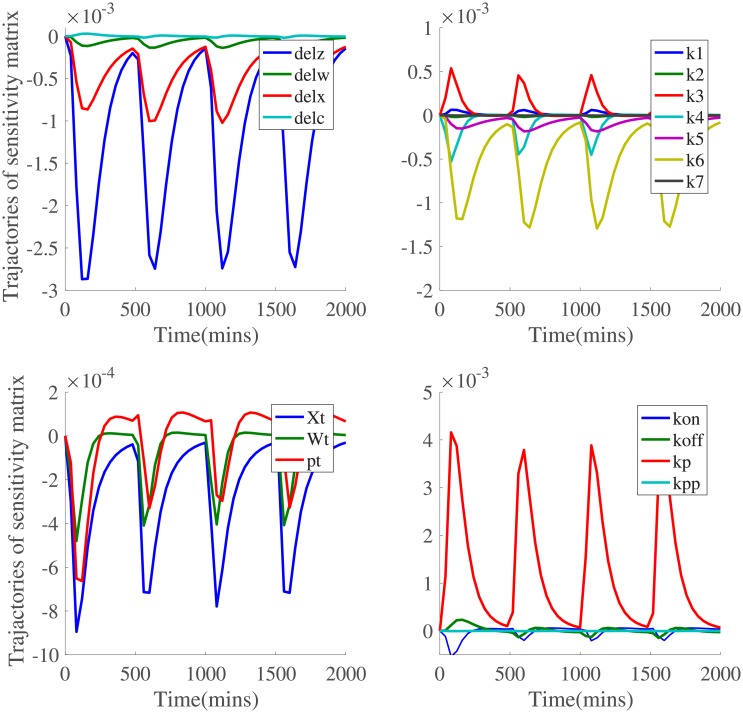
Sensitivity analysis of model parameters for buffered gene network. Among these 23 kinetic parameters, sensitivity matrices of 18 model parameters are plotted to reflect the various levels of influence.

For this buffered network, sensitivity analysis is performed to detect key parameters that have significant influence on the output behavior. Among 23 model parameters in the buffered system, this study selects 18 parameters and computes the corresponding sensitivity matrices. For buffered gene networks, the trajectories of sensitivity matrices of 18 kinetic parameters that change over time are depicted in Fig 8.

**Fig 9:**
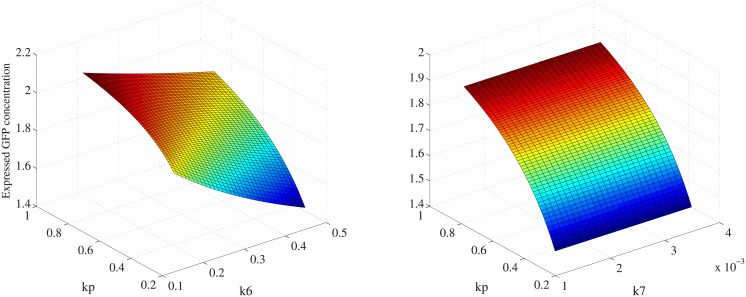
Quantitative influence of kinetic parameters on system output. Kinetic parameters with various sensitivity levels have different degree of influence on system output, which correspond to the expressed level of reporter protein *GFP* in the synthetic genetic network.

From basic sensitivity analysis, kinetic parameters *δ*_*z*_, *k*_6_, *k*_*p*_ are considered kinetic parameters with highest level of sensitivity. Chemical reactions involved in genetic networks can be reversible. Each reaction has two parameters related with production rates. Perturbations of production rates are more likely to alter the output behaviors of genetic circuits. It is noted that *δ*_*z*_, *k*_6_ have negative sensitivity while *k*_*p*_ has positive sensitivity. Subsequent analysis focus on quantitative impact of those kinetic parameters with high sensitivity levels on the system output. Based on trajectories of sensitivity matrices in Fig 8, parameters *k*_*p*_ and *k*_6_ have positive and negative sensitivity respectively. Furthermore, ±50% numerical perturbations have been introduced to kinetic parameters *k*_*p*_, *k*_*pp*_, *k*_6_ and *k*_7_ that have different degrees of influence on the system output. Quantitative influences on the system output *GFP* are analyzed in three-dimensions, shown in Fig 9.

Influence of kinetic parameters with various sensitivity levels are analyzed. Parameters including *k*_*p*_ and *k*_6_ with high sensitivity have relatively strong impact on the system output. In Fig 9(a), parameters *k*_*p*_ and *k*_6_ have exhibited opposite impacts on the system output, i.e. the expressed *GFP* concentration. When the value of *k*_*p*_ increases, the expressed *GFP* level is promoted as a consequence. It is observed from Fig 9(b) that variation of *k*_*p*_ has significant impact on output behaviors while *k*_7_ has a limited power to change expression behaviors of reporter protein *GFP*.

**Fig 10:**
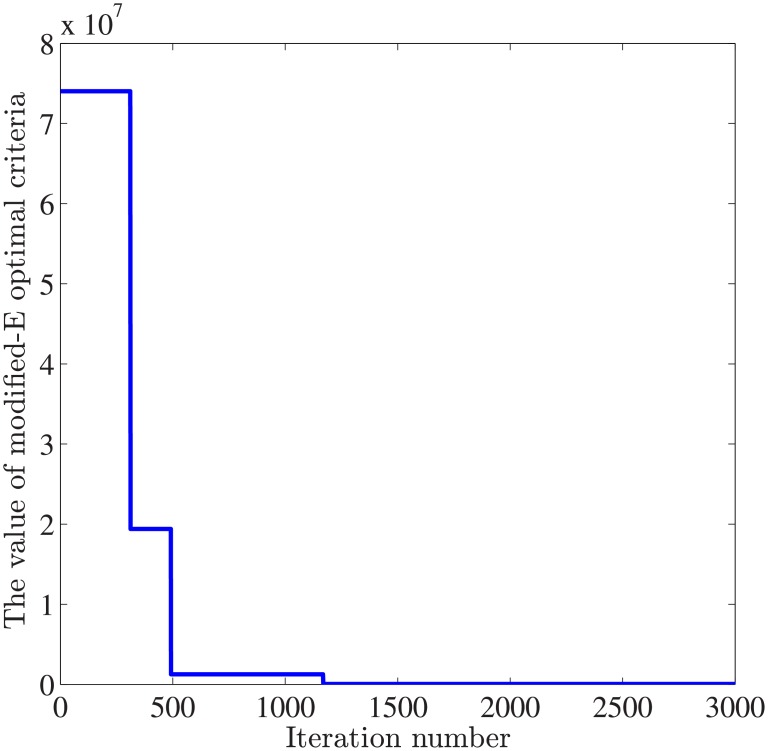
Trajectories of the modified E-optimality criteria using HS-OED algorithm. In the outer loop, the value of modified-E optimal criteria decreases as the value of fitness function is minimized by the proposed HS-OED method.

In order to compute the modified-E optimal criteria and select the optimal input magnitude, the proposed two-stage to calculate the best input level u* and the parameter vector p* in double nested loops. In the outer iteration loop, the HS algorithm parameters are settled as *uIter* = 3000, *HMS* = 15, *HMSu* = 10, *HMCR* = 0.8. According to the previous analysis of input-response relations, the feasible interval for input level is selected as [1,30]*μM*. In the second stage of optimization, the maximum number of iterations in the inner loop is *pIter* = 5000. Other parameters are the same with that in outer loop. The modified E-optimality criteria and cost function *cfun*1(*u*, p*) are minimized in the outer loop. In Fig 10, the trajectories of modified E-optimality criteria under five inputs monotonically decreases and converges to a domain around zero.

During the first stage of optimization, the error value reaches the minimum when the iteration number exceeds 2000. This study focuses on keeping a balance between experimental cost and estimation accuracy in identification of synthetic gene networks. After initial curve fitting and parameter refinement, the optimal parameter vector for the buffered system is estimated using the proposed two-stage optimization approach.

**Fig 11:**
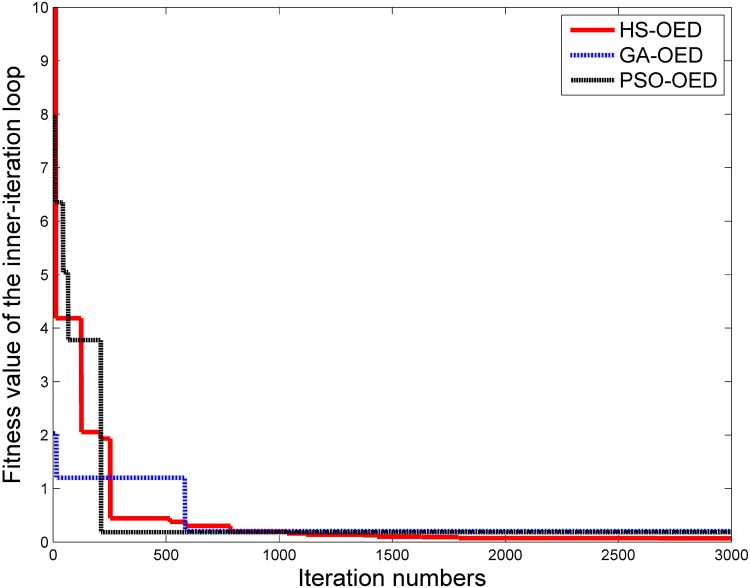
Fitness trajectories of harmony search denoted by HS-OED and two heuristic optimization approaches in the inner iteration loop of OED. The HS-OED algorithm has reduced computational time than GA and PSO algorithm, and obtaining lower output errors in estimating parameter vectors.

In the two step optimization, the outer loop determines the optimal input while the inner loop applies the designed input to estimate parameter vector. In order to compare the proposed HS-based OED with other approaches, various optimization are compared in the inner iteration loop, where the fitness function denote estimation error. Heuristic optimization approaches including particle swarm optimization(PSO) and genetic algorithm(GA) are able to perform the task of optimal experimental design with suitable fitness functions [41, 42]. Under the framework of deterministic modeling, GA and PSO algorithms have been applied to minimize the fitness function value, thus estimating optimal parameter vectors. For GA algorithm, the population size is 50, the mutation and crossover rate are settled as 0.8 and 0.2, which correspond to *HMCR* and *PAR* in HS algorithm. For PSO algorithm, the weight is 1 and coefficients are *c*_1_ = *c*_2_ = 1. Trajectories of fitness function provided by GA,PSO and HS-OED algorithm are compared by Fig 11.

In Fig 11, the proposed HS-OED algorithm has shown similar convergence patterns compared with GA and PSO optimization approaches. Standard PSO method usually suffers from premature convergence problem due to loss of diversity in solution search. Experiments of optimal experimental design are performed by the MATLAB software on a PC with Intel i5-3320M and 8GB RAM. After four replicates, the average computation time of 3000 iterations for HS-OED algorithm is 428 seconds. While average computational time of GA and PSO-based OED for 3000 iterations are 3137 and 7378 seconds. In addition, the average best fitness value of HS-OED is computed as 0.0332, which is significantly lower than 0.207 and 0.1873 obtained by GA and PSO-based OED respectively. In this case, HS-OED algorithm provides a feasible and efficient solution to improve optimization ability.

To evaluate the performance of these optimal identification methods, the proposed HS-OED algorithm is further compared with other two methods denoted by PSO-OED and GA-OED in accuracy evaluation. After parameter estimation, estimation accuracy is analyzed based on the value of Crámer-Rao lower bound(CRLB). In estimation analysis, the CRLB values of kinetic parameters *k*_*i*_(i = 1,…,5) are compared in Table 4.

**Table 4:** Comparison of lower bounds in estimating kinetic parameters *k*_*i*_ obtained by HS-OED algorithm and GA,PSO-based OED methods. These indexes are obtained in numerical simulation.

Methods	*k*_1_	*k*_2_	*k*_3_	*k*_4_	*k*_5_
PSO-OED	270.4	1.283	32.7	3.848	0.250
GA-OED	17.2	0.208	4.337	0.667	0.319
HS-OED	4.62	0.086	1.432	0.375	0.197

**Fig 12:**
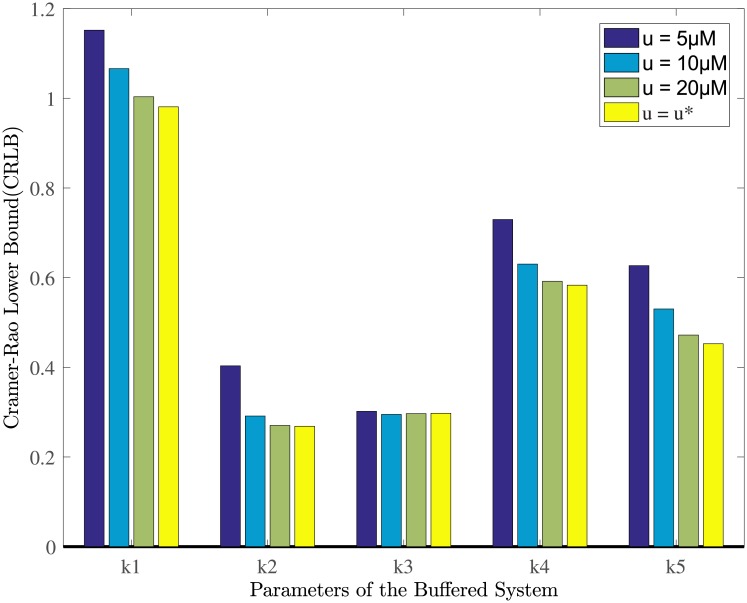
Crámer-Rao lower bound(CRLB) of kinetic parameters *k*_1_, *k*_2_, *k*_3_, *k*_4_ and *k*_5_ of the buffered system. Compared with three input levels i.e. *u* = 5*μM*, 10*μM*, 20*μM*, the optimal input u* is able to reduce the values of CRLB for specific parameters.

Those parameters with higher CRLB values are considered to have higher degree of uncertainty during modeling. Accuracy indexes computed by HS-OED algorithm are significantly lower than that of other two OED methods. And GA-OED has provided more superior performance than PSO-OED. Using experimentally measured step response data, the CRLB for estimated parameters *k*_1_, *k*_2_ are computed as 1.486 and 0.185, which are higher than that of *k*_*on*_, *k*_*off*_ in the unbuffered system. For the buffered system, the CRLB for production rates of *k*_3_, *k*_4_ are computed as 1.432 and 0.375. The kinetic parameter *k*_*g*_ has the highest CRLB value of 2.2767, indicating that it has the highest level of uncertainty. With the purpose of illustrating the advantage of optimal input u*, the values of CRLB for five parameters under *u* = 5*μM*, 10*μM*, 20*μM* and u* are compared in Fig 12.

**Fig 13:**
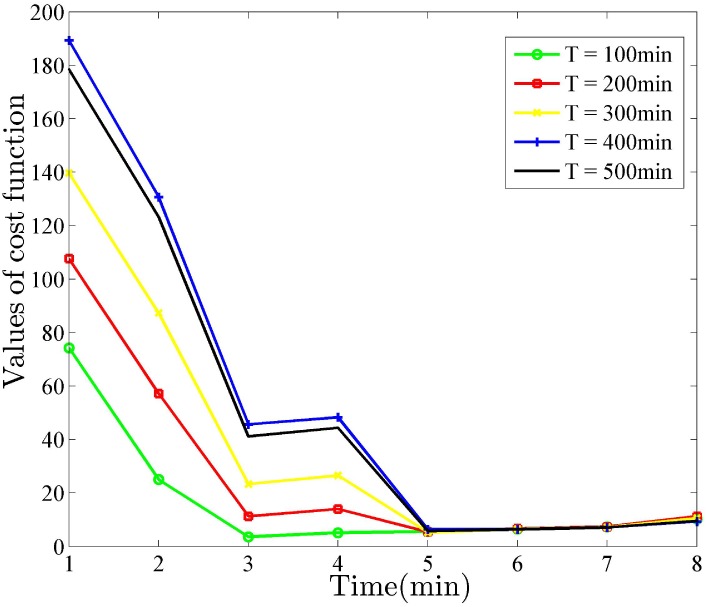
Cost trajectories of five periodic inputs for buffered system. Five trajectories of cost function converge to the point *N* = 50, indicating the amount of measurements meet the requirement of modeling.

The number of measured time points *n*_*m*_ is another crucial factor that influence the modeling quality. After reaching a specific threshold, the improvement of model accuracy brought by increased *n*_*m*_ become limited. Meanwhile, the variance of parameter estimation decline to decrease and stays a level that is beyond that of CRLB. The optimal identification method tries to control the experimental cost without loss of much modeling quality. After simulation experiments and comparison, the penalty coefficient λ in cost function is tuned for specific gene networks to achieve a tradeoff. To find the suitable number of measurements, the experiments apply *n*_*m*_ as the independent variable and calculate the value of cost function that has two penalty terms. Trajectories of cost function are recorded with the increasing iterations, shown in Fig 13.

The step is set as 10 and the penalty coefficient λ for measurement cost is suggested as 0.8. It is observed from Fig 13 that five trajectories of cost function meet together at the point which correspond to *n*_*m*_ = 50 measured time points. This mean that the sample size *n*_*m*_ = 50 can be regarded as a suitable number of measured time points for buffered system. Considering the limited number of time points single experiment, several perturbation experiments under different conditions can be performed to collect enough number of measurements.

**Fig 14:**
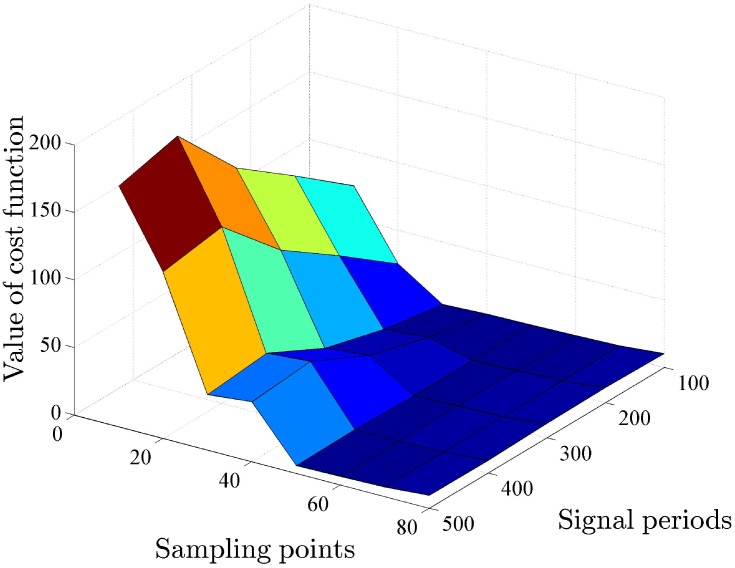
The three-dimensional surface of designed cost function under five square inputs. The optimal identification algorithm picks *n*_*m*_ = 50 as the suitable number of measured time points.

As visualizing the trajectories of cost function provide limited information about optimal identification, the three-dimensional surface depicts a broader view of this process, shown in Fig 14.

The measurement cost is reflected by the number of observed data points *n*_*m*_ which is proportional to resources spent in experiments. The optimal identification method increases the number of measured time points at a step of 10. In order to reach the same normalized accuracy level, which can be denoted by *CRLB*/*n*_*m*_, the necessary number of measured points in buffered system is approximately 67% higher than that in unbuffered system. since *n*_*m*_ and *n*_*p*_ equal to 50 and 23 in two systems. For buffered gene circuit, the value of penalty term that represents the measurement cost (*n*_*m*_ − *n*_*p*_)/*n*_*m*_ equal to 0.54, which multiplies a penalty coefficient λ. The proposed optimal identification is effective to pursue a balance between two factors in deterministic modeling of gene circuits.

Experimental outcomes with two synthetic gene circuits indicate the parameter refinement is able to improve the model quality as well as to reduce the uncertainty. Different with traditional single optimality criteria, the propose optimal identification suggest not to increase the measured points of output when the profit of increasing measurements is not significant. Computational burden of this nested iteration framework is still huge, especially for complex synthetic gene networks. Incorporating biological prior knowledge in the constraints conditions will be beneficial to reduce the viable region.

## 4 Conclusion

In order to guide the modular construction of synthetic gene networks, a novel optimal identification method that provides accurate predictive models at a low experimental cost is proposed. For synthetic gene networks, optimal experiment design(OED) is feasible and effective to design an appropriate input level through maximizing information content related optimal criteria. In this case, this paper proposes a harmony search-based OED(HS-OED) approach and designs a two-stage optimization. Optimal input signals and parameter vectors are computed by this two-stage optimization framework. The first contribution of this paper is to apply harmony search strategy instead of brute screening search to improve the accuracy and efficiency in searching optimal inputs. Secondly, the designed cost function can be used to select an appropriate number of data points collected for computational modeling of gene circuits. Simulation and experimental analysis indicate the proposed HS-OED method can obtain accurate model parameters than established heuristic algorithms based OED method, with enhanced estimation accuracy. Furthermore, the amount of measured data points are reduced to offer a low-cost identification solution for synthetic gene networks.

## Appendix

In the parameter estimation experiments, kinetic parameters of two synthetic gene networks are computed based on the mechanistic mathematical models. The ODE model for unbuffered system consists of 3 equations, shown as follows.

$$\begin{array}{c} {\dot{X}}_{m}=k_{m}\frac{{\left(DOX\right)}^{n_{1}}}{K_{dox}+{\left(DOX\right)}^{n_{1}}}-\delta_{M}X_{m}-k_{on}X_{m}\left(p_{T}-C_{m}\right)+k_{off}C_{m}+\delta_{c}C_{m}\\ {\dot{C}}_{m}=k_{on}X_{m}\left(p_{T}-C_{m}\right)-k_{off}C_{m}-\delta_{c}C_{m}\\ {\dot{\hat{G}}}_{m}=k_{sgfp}+k_{g}\frac{{\left(X_{m}\right)}^{n_{2}}}{K_{gfp}+{\left(X_{m}\right)}^{n_{2}}}-\delta_{G}{\hat{G}}_{m} \end{array}$$

where *X*_*m*_ and ${\hat{G}}_{m}$ denote abundance of *SKN*7*m* and reporter protein GFP respectively, *n*_1_, *n*_2_ are Hill coefficients. The ODE system for buffered system consists of 10 equations and 23 kinetic parameters. Assume *Z* denotes STAT5-HKRR fusion, *W*, *X* represent YPD1 and SKN7, $\hat{G}$ is the reporter protein *GFP*. Thus the ODE system for buffered genetic system can be described as follows:

$$\begin{array}{c} \dot{Z}=k_{m}\frac{{\left(DOX\right)}^{n_{1}}}{K_{dox}+{\left(DOX\right)}^{n_{1}}}-\delta_{Z}Z-k_{2}W^{*}Z+k_{1}Z^{*}\left(W_{T}-W^{*}\right)-k_{p}Z+k_{p}^{\prime }Z^{*}\\ {\dot{Z}}^{*}=-k_{1}Z^{*}\left(W_{T}-W^{*}\right)+k_{2}W^{*}Z+k_{p}Z-k_{p}^{\prime }Z^{*}-\delta_{Z}Z^{*}\\ {\dot{W}}_{T}=k_{w}-\delta_{W}W_{T}\\ {\dot{W}}^{*}=k_{1}Z^{*}\left(W_{T}-W^{*}\right)-k_{2}W^{*}Z-k_{3}\left(X_{T}-X^{*}-X^{**}-C^{*}-C^{**}\right)W^{*}\\ \;\;+k_{4}X^{*}\left(W_{T}-W^{*}\right)-\delta_{W}W^{*}-k_{3}X^{*}W^{*}+k_{4}X^{**}\left(W_{T}-W^{*}\right)-k_{7}W^{*}\\ {\dot{X}}_{T}=k_{x}-\delta_{X}X_{T}\\ {\dot{X}}^{*}=k_{3}\left(X_{T}-X^{*}-X^{**}-C^{*}-C^{**}\right)W^{*}-k_{4}X^{*}\left(W_{T}-W^{*}\right)-\delta_{X}X^{*}\\ \;\;-k_{3}X^{*}W^{*}+k_{4}X^{**}\left(W_{T}-W^{*}\right)-k_{5}X^{*}+k_{6}X^{**}+r_{1}\\ {\dot{X}}^{**}=k_{3}X^{*}W^{*}-k_{4}X^{**}\left(W_{T}-W^{*}\right)-k_{6}X^{**}-\delta_{X}X^{**}+r_{2}\\ {\dot{C}}^{*}=k_{on}X^{*}\left(p_{T}-C^{*}-C^{**}\right)-k_{off}C^{*}-\delta_{C}C^{*}\\ {\dot{C}}^{**}=k_{on}X^{**}\left(p_{T}-C^{*}-C^{**}\right)-k_{off}C^{**}-\delta_{C}C^{**}\\ \dot{\hat{G}}=k_{sgfp}+k_{g}\frac{{\left(X^{**}\right)}^{n_{2}}}{K_{gfp}+{\left(X^{**}\right)}^{n_{2}}}-\delta_{G}\hat{G} \end{array}$$

where *G* represents the expression level of reporter protein *GFP*, parameters *k*_*m*_, *k*_*g*_ are maximum activated protein production rates, *K*_*dox*_, *K*_*gfp*_ are respective *K*_*d*_ for Hill equations, *k*_*w*_, *k*_*x*_ are production rates for *YPD*1 and *SKN*7 respectively. Kinetic parameters *k*_*i*_(*i* = 1, 2, 3, 4) are constrained to 1-50 [*μM*]^−1^. Based on prior knowledge, the conditions *k*_1_ ≥ *k*_3_, *k*_2_ ≥ *k*_4_ are used as constraints during computational modeling. Retroactivity between modules are denoted by *r*_1_, *r*_2_ which will attenuate the response behaviors of synthetic gene networks.
